# Dextromethorphan for Restless Legs Syndrome: Testing the Glutamate Theory

**DOI:** 10.7759/cureus.105315

**Published:** 2026-03-16

**Authors:** Melissa Khalil, Estefany Garces Delgado, Brynn Dredla, Joseph Cheung, Pablo Castillo

**Affiliations:** 1 Sleep Medicine, James H. Quillen VA Medical Center, Johnson City, USA; 2 Sleep Medicine, Mayo Clinic, Jacksonville, USA

**Keywords:** adenosine pathways, dextromethorphan, drug repurposing, glutamatergic signaling, nmda receptor antagonist, restless legs syndrome, sleep disorders

## Abstract

Restless legs syndrome (RLS) is a common neurological sensorimotor disorder associated with hyperarousal, an urge to move the legs, and significant sleep disruption. While dopaminergic dysfunction and iron deficiency have been implicated in its pathophysiology, emerging evidence also suggests roles for adenosinergic and glutamatergic systems. We report the case of a 45-year-old woman with severe, treatment-refractory RLS who experienced substantial and sustained symptom improvement with oral dextromethorphan (DXM), initially taken for an unrelated upper respiratory infection. Her prior therapies included dopamine agonists, which resulted in augmentation, and gabapentin, which was poorly tolerated. Upon initiating over-the-counter DXM at 30 mg nightly, the patient reported near-complete resolution of RLS symptoms, reflected by a mild to moderate International Restless Legs Scale (IRLS) score of 12 and a significant reduction in insomnia severity. DXM, a noncompetitive NMDA receptor antagonist with anti-inflammatory properties, may exert therapeutic effects through modulation of central glutamatergic activity and enhancement of adenosine tone. There is limited literature supporting the use of DXM for the treatment of RLS, and further research is needed to validate its potential as a therapeutic option. These findings underscore the importance of exploring novel, mechanism-based approaches for RLS treatment, particularly in patients who do not respond to standard therapies.

## Introduction

Restless legs syndrome (RLS), also known as Willis-Ekbom disease, is a common neurologic sensorimotor disorder characterized by unpleasant sensations during inactivity, associated with a hyperarousal state and an urge to move the legs [1]. The prevalence of clinically significant RLS is approximately 3% of US adults [1]. The pathophysiology of RLS is not fully understood; however, studies have demonstrated alterations in several neurotransmitter systems (including dopamine, glutamate, adenosine, and endogenous opioids), as well as abnormalities in iron metabolism, inflammatory pathways, and genetic factors [2,3]. We report a patient with severe RLS who failed multiple treatments and whose symptoms were substantially improved with oral dextromethorphan (DXM) as monotherapy.

This article was previously presented as an abstract at the Associated Professional Sleep Societies (APSS) meeting in June 2024.

## Case presentation

**Figure 1:**
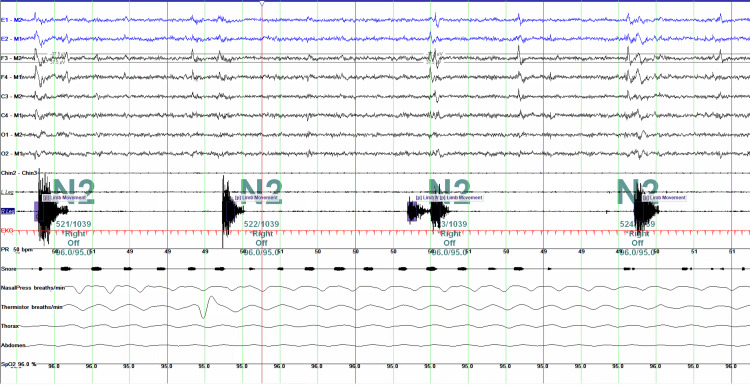
Polysomnogram (PSG) excerpt demonstrating periodic limb movements of sleep (PLMS) during non–rapid eye movement (NREM) stage N2 Displayed channels include electroencephalography (e.g., C4–M1, F4–M1, O2–M1), electrooculography (e.g., E1-M2), chin electromyography (EMG), electrocardiography (EKG) with heart rate or beats per minute (BPM), airflow (nasal/oral), respiratory effort (chest and abdominal belts), oxygen saturation (SpO₂), and limb movement (leg EMG). Leg EMG channels show recurrent bursts of high-frequency, high-amplitude activity consistent with periodic limb movements (PLMs) highlighted in purple. This tracing demonstrates repetitive muscle activation in the lower extremities during otherwise normal N2 sleep, with the respiratory and EKG channels showing stable breathing and a regular heart rate, respectively.

A 45-year-old woman presented with more than a decade of symptoms consistent with RLS associated with sleep-onset insomnia. She described “growing pains” in her legs as a child, and her family history was significant for RLS affecting her father and 2 siblings. A diagnosis of definite RLS was established based on the polysomnographic findings shown in Figure 1 and fulfillment of the diagnostic criteria defined by the International Restless Legs Syndrome Study Group, with all essential features present [4].

The severity of RLS was assessed by nightly occurrence and an Insomnia Severity Index score of 25, consistent with severe clinical insomnia, as well as an elevated International Restless Legs Syndrome (IRLS) score of 30, consistent with severe symptom burden. There was no evidence of peripheral neuropathy on the neurologic examination. She was not being treated with antidepressants or dopamine antagonists. Her estimated total caffeine intake was approximately 290 mg caffeine per day.

The patient had previously been treated with different dopamine agonists, including pramipexole and ropinirole at recommended standard doses, which led to augmentation of her symptoms. Laboratory tests revealed her ferritin level was adequate at 120 ng/mL (N=30-300 ng/mL), and her magnesium level was also normal. Polysomnography excluded additional sleep-disordered breathing but revealed a periodic limb movement-arousal index of 20 [5]. The patient was advised to discontinue all sources of caffeine to help improve her insomnia, which she did following her initial appointment. She initially declined treatment with opioid medications. A trial of gabapentin, an α-2-δ calcium channel ligand, was initiated at a dose of 300 mg nightly and titrated up to 800 mg. At that point, the patient developed side effects, prompting a dose reduction to 600 mg nightly, which provided only partial relief of symptoms.

While using over-the-counter DXM for an acute cough related to an upper respiratory infection, the patient reported marked subjective improvement of her RLS symptoms. Because of this improvement, she discontinued gabapentin after one week of using DXM. On her own, the patient continued to use oral DXM at 30 mg before her bedtime for approximately 6 months, with consistent improvement of RLS symptoms. Her Insomnia Severity Index score was 10, consistent with no significant insomnia, and her IRLS scale score was 12, indicating mild to moderate symptom burden.

At her follow-up appointment, the patient requested an alternative medication since DXM was not covered by her insurance. DXM was tapered, resulting in the recurrence of symptoms. The patient was subsequently started on tramadol monotherapy, titrated to 100 mg at bedtime, which successfully controlled her RLS symptoms. The patient declined follow-up polysomnography to reassess periodic limb movement burden.

## Discussion

The pathophysiology of RLS is complex and only partially understood. Iron deficiency impairs dopamine synthesis by affecting tyrosine hydroxylase activity, an iron-dependent enzyme crucial for dopamine production [6]. Additionally, animal models of brain iron deficiency demonstrated that cortico-striatal glutamatergic terminals exhibit hypersensitivity [7]. Iron deficiency also causes downregulation of adenosine A1 receptors (A1R) in the cortex and striatum, along with functional upregulation of adenosine A2A receptors (A2AR) in the striatum. These changes enhance the sensitivity of glutamatergic cortico-striatal terminals, leading to heightened cortical excitability and hyperarousal, as well as motor hyperexcitability [8]. The rationale for targeting possible downregulated endogenous adenosine tone in patients with RLS is supported by recent results obtained with dipyridamole, an inhibitor of equilibrative nucleoside transporter-1 (ENT1), which increases extracellular availability of adenosine [3].

Caffeine impacts the adenosine pathways in the central nervous system by acting as a non-selective antagonist of adenosine receptors, primarily A1 and A2A receptors. Adenosine typically promotes sleep and relaxation by inhibiting neuronal activity. When caffeine blocks these receptors, it prevents adenosine from exerting its calming effects, leading to increased arousal and wakefulness [9].

In the context of RLS, this antagonism can exacerbate symptoms [10]. To enhance the endogenous adenosine tone, the patient was asked to stop all sources of caffeine, including tea and dark chocolate. It is difficult to determine the extent to which caffeine cessation contributed to the improvement of RLS symptoms, and it may represent a potential confounder. However, the persistent response observed following initiation of DXM may suggest an underlying pharmacologic effect beyond the placebo effect or from caffeine elimination alone.

Enhanced glutamatergic activity may play a role in both the sensorimotor symptoms and hyperarousal state [2]. In fact, α-2-δ calcium channel ligands, a class of RLS treatment, work by inhibiting the presynaptic glutamate release [2]. Furthermore, central nervous system iron deficiency may lead to increased glutamatergic tone, with subsequent hyperarousal state, in genetically vulnerable individuals [2,11,12].

Given the hyperglutamatergic state implicated in RLS pathophysiology, N‑methyl‑D‑aspartate (NMDA) antagonism may contribute to symptom relief by dampening glutamate-mediated excitation. Ketamine, an NMDA receptor antagonist, inhibits glutamate‑mediated activation of the NMDA receptor and reduces presynaptic glutamate release. It has been reported as an effective treatment in a small number of patients with RLS [13]. In vitro studies have shown that tramadol and its metabolite 0-desmethyltramadol (M1 metabolite) also inhibit NMDA receptors in a concentration-dependent, noncompetitive manner, though this effect occurs at relatively high concentrations [14]. Due to their abuse potential, opioids are typically reserved for patients who have not responded to other pharmacologic therapies [15].

DXM is another oral NMDA receptor antagonist; however, there are limited data in the medical literature supporting the use of DXM as treatment for RLS. At therapeutic doses, DXM occasionally causes drowsiness, dizziness, and gastrointestinal disturbances. However, at supratherapeutic doses, a broader toxidrome emerges, including ataxia, hyperexcitability, dystonia, coma, psychosis, dysautonomia, and urticarial rash. At doses exceeding 2 mg/kg (or >1500 mg/day), it produces dissociative effects similar to ketamine due to NMDA receptor antagonism. Dextromethorphan also inhibits serotonin reuptake and can cause serotonin syndrome, particularly when combined with other serotonergic agents [16].

Furthermore, chronic inflammatory markers may be associated with RLS [17]. In this context, Chen et al. reported that DXM exerts anti-inflammatory and immunomodulatory effects, likely through the inhibition of proinflammatory cytokine expression and autoantibody production [18].

## Conclusions

The pathophysiology of RLS is partially understood. An enhanced glutamatergic activity has been reported to be a contributing factor to the hyperarousal state and the sensorimotor symptoms that patients with RLS experience. Limited data have shown that ketamine, an NMDA receptor antagonist, is effective in treating RLS. Dextromethorphan, another NMDA receptor antagonist, has the same inhibiting effect on glutamate. We report the case of a middle-aged woman with severe RLS who had dramatic and sustained improvement in her symptoms while taking DXM for an unrelated upper respiratory infection. However, the efficacy and safety of long-term use of DXM for the treatment of RLS are uncertain. We propose that there is some limited biological plausibility to support investigating DXM as a potential treatment for RLS. Clinical trials are needed to validate the efficacy of DXM for this use.
